# Bayesian estimation of explained variance in ANOVA designs

**DOI:** 10.1111/stan.12173

**Published:** 2019-03-27

**Authors:** Maarten Marsman, Lourens Waldorp, Fabian Dablander, Eric‐Jan Wagenmakers

**Affiliations:** ^1^ University of Amsterdam Amsterdam The Netherlands; ^2^ University of Tübingen Tübingen Germany

**Keywords:** analysis of variance, credible interval, effect size

## Abstract

We propose to use the squared multiple correlation coefficient as an effect size measure for experimental analysis‐of‐variance designs and to use Bayesian methods to estimate its posterior distribution. We provide the expressions for the squared multiple, semipartial, and partial correlation coefficients corresponding to four commonly used analysis‐of‐variance designs and illustrate our contribution with two worked examples.

## 1 INTRODUCTION

In the empirical sciences, researchers are commonly advised to report effect size (ES) interval estimates to complement the use of statistical hypothesis testing (e.g., Loftus, 1996; Cohen, 1992, 1994; Cumming, 2014). This is particularly true for psychology, where the need for reporting ES estimates is stressed in publication guidelines of several organizations and scientific journals (see Kelley & Preacher, 2012; Peng, Chen, Chiang, & Chiang, 2013 for recent surveys), but it also applies to other fields of science, such as biology (Nakagawa & Cuthill, 2007), genetics (Park et al., 2010), and marketing research (Fern & Monroe, 1996). Clearly, the use and interpretation of ES interval estimates is of practical importance and continues to attract much research interest (e.g., Huberty, 2002; Robinson, Whittaker, Williams, & Beretvas, 2003; Alhija & Levy, 2008; Fritz, Scherndl, & Kühberger, 2012; Grissom & Kim, 2012; Lakens, 2013; Fritz, Morris, & Richler, 2012).

Despite the continued interest in ES interval estimation, we have two concerns as to their development for the analysis of variance (ANOVA) model, which is arguably the most often applied statistical model across the empirical sciences. Our first concern is that ES measures for ANOVA models are often defined in a somewhat ad hoc manner and follow what Kelley and Preacher (2012, p. 139) call a *definition‐by‐example* approach, making it difficult to generalize the ES measures beyond the specific examples given. One case in point is the *ω* ^2^ measure for the two‐way ANOVA (Hays, 1963, p. 406) and the subsequent difficulty of generalizing it to a partial *ω* ^2^ measure (Keren & Lewis, 1979). Similar issues have been reported for the commonly used *η* ^2^ measure (Cohen, 1973; Kennedy, 1970; Levine & Hullet, 2002; Pierce, Block, & Aguinis, 2004). Thus, what is lacking is a general framework within which ES measures such as *ω* ^2^ and *η* ^2^ can be derived.

Our second concern is that ES interval estimation for ANOVA models has been treated almost exclusively from a classical perspective. Despite their popularity, classical confidence intervals (CIs) are often misunderstood and misinterpreted (Hoekstra, Morey, Rouder, & Wagenmakers, 2014; Morey, Hoekstra, Rouder, Lee, & Wagenmakers, 2016). Furthermore, Morey et al. (2016) demonstrated that current CI procedures for *ω* ^2^ are particularly problematic, as they can produce empty intervals and involve post hoc manual truncation at the boundary of the parameter space. In contrast to the classical CIs, Bayesian credible intervals are more straightforward to interpret (Wagenmakers, Morey, & Lee, 2016). In addition, credible intervals do not have the anomalies that beset some of the classical ES intervals for ANOVA designs. Despite their added value, however, Bayesian methods of ES estimation for ANOVA models have so far received little attention.

In light of these concerns, our goals are twofold. Our primary goal is to show that the theory on squared multiple correlations *ρ* ^2^—well established for linear regression models—also provides a unifying framework for ES measures in ANOVA designs. Several ES measures that are commonly reported in the ANOVA literature (e.g., *ω* ^2^, *η* ^2^, partial *ω* ^2^, and partial *η* ^2^) will be shown to be special cases of *ρ* ^2^. Although the connection between ES measures such as *ω* ^2^ and *η* ^2^ for the ANOVA model and the squared multiple correlations *ρ* ^2^ for the linear regression model has been noted before (e.g., Keren & Lewis, 1979; Maxwell, Camp, & Arvey, 1981; Sechrest & Yeaton, 1982; Cohen, Cohen, West, & Aiken, 2003), little is known about the assumptions that underpin this relation. We will detail these assumptions and indicate the relation for several commonly used ANOVA designs, thereby providing a formal basis for the development of ES measures for the ANOVA model.

Our secondary goal is to estimate the squared multiple correlations using Bayesian methods. Although the issue of Bayesian estimation of multiple correlations has received considerable attention in the context of linear regression (see, for instance, Geisser, 1965; Press & Zellner, 1978; Tiwari, Chib, & Jammalamadaka, 1989; Gross, 2000), it has received almost no attention in the context of ANOVA models. One notable exception is the work of Gelman and Pardoe (2006), which focused on hierarchical extensions of the classical ANOVA model (see also Gelman, 2005). While the approach of Gelman and Pardoe (2006) was based on a sample definition of *ρ* ^2^ (i.e., *R* ^2^), we instead derive the *population* expressions for *ρ* ^2^. The resulting expressions are found to be functions of the ANOVA model parameters, such that *ρ* ^2^ can be estimated from the posterior distribution of the ANOVA model parameters.

Below, we first discuss the general background, followed by a more targeted treatment of four popular ANOVA designs. We end by presenting two worked examples.

## 2 GENERAL BACKGROUND

Let ${\left(y,\;x^{T}\right)}^{T}$ denote a (column) vector of variables with covariance matrix

(1) $$\Sigma =\left(\begin{array}{cc} \mathrm{Var}(y) & \text{Cov}(y,\;{\text{x}}^{T})\\ \text{Cov}(\text{x},\;y) & \mathrm{Var}(\text{x}) \end{array}\right)=\left(\begin{array}{cc} \Psi & \Delta^{T}\\ \Delta & \Gamma \end{array}\right).$$

We will refer to variable *y* as a dependent variable and the (vector of) variable(s) **x** as the independent variable(s). Given the covariance matrix **Σ**, we can express the relation between the dependent variable *y* and the independent variables **x** using the squared multiple correlation coefficient (Tate, 1966; Cohen, 1982; Mudholkar, 2014), as follows:

(2) $$\rho^{2}=\rho_{y\;x}^{2}=1-\frac{\left|\Sigma \right|}{\left|\Psi ||\Gamma \right|}=1-\frac{|\Gamma |\left(\Psi -\Delta^{T}\Gamma^{-1}\Delta \right)}{\Psi |\Gamma |}=\frac{\Delta^{T}\Gamma^{-1}\Delta }{\Psi },$$

provided that the covariance matrix **Γ**=Var(**x**) is nonsingular and that Ψ = Var( *y*) > 0. The squared multiple correlation coefficient *ρ* ^2^ is the maximum squared correlation that can be obtained between a single dependent variable *y* and any linear combination of the set of independent variables **x** (e.g., Anderson, 1958, p. 88).

Similar to most of the ES measures that have been proposed for the ANOVA model, the squared multiple correlation coefficient *ρ* ^2^, as defined in Equation (2), is a so‐called *proportional reduction in error* (PRE) measure (Reynolds, 1977). In general, a PRE measure expresses the proportion of the variance in an outcome *y* that is attributed to the independent variables **x**. Here, we treat the assignment in a design D as a random variable, and we decompose the variance of the dependent variable as follows:

(3) Var(y)=Var(E(y∣x))+E(Var(y∣x)).

Because the ANOVA model assumes homoscedasticity, the design affects only the conditional expectations (means) of the dependent variable *y*. In other words, Var(E( *y*∣**x**)) is the variance that can be attributed to random assignment in the design, and E(Var( *y*∣**x**)) is the residual variation that is constant across the cells in the design D. As a result, we can reexpress Equation (3) as

$$\sigma_{Y}^{2}=\sigma_{D}^{2}+\sigma_{E}^{2},$$

where $\sigma_{D}^{2}=\mathrm{Var}(E(y|x))$ denotes the design (or assignment) variance and $\sigma_{E}^{2}=E(\mathrm{Var}(y|x))$ denotes the error variance. In this form, Equation (3) is used in the ANOVA literature to define PRE measures as

(4) $$1-\frac{\sigma_{E}^{2}}{\sigma_{Y}^{2}}=\frac{\sigma_{D}^{2}}{\sigma_{Y}^{2}}=\frac{\sigma_{D}^{2}}{\sigma_{D}^{2}+\sigma_{E}^{2}}.$$

ANOVA ES measures defined in this manner include *ω* ^2^ (Hays, 1963, p. 382), *η* ^2^ (Hays, 1963, p. 547), and the intraclass correlation coefficient $\rho_{I}^{2}$ (Hays, 1963, p. 424). In case of an ANOVA design with a single fixed factor, both *ω* ^2^ and *η* ^2^ specify or estimate $\sigma_{D}^{2}$ as the sum of squares that are attributed uniquely to the fixed factor and $\sigma_{E}^{2}$ as the residual variance (see Analysis I). When the factor is random, $\sigma_{D}^{2}$ can be taken to be the variance of the random factor to define $\rho_{I}^{2}$ (see Analysis IV).

The definition of *ρ* ^2^ in Equation (2) is of the same form as Equation (4)—note that $\sigma_{Y}^{2}=\Psi$ and $\sigma_{D}^{2}=\Delta^{T}\Gamma^{-1}\Delta$. In order to apply Equation (2) to ANOVA, we have to define the joint model *p*( *y*,**x**), with **x** encoding the ANOVA design, from which we can derive **Σ**. To this aim, we first specify the ANOVA model as an ordinary linear regression model, utilizing dummy variables to encode the fixed effects (Jennings, 1967; Cohen, 1968). That is, for the fixed effects, we introduce indicator variables *x*_*i*_, with

$$x_{i}=\left\{\begin{array}{cc} 1,\; & \text{if subject is in group}\;i\\ 0,\; & \text{if subject is not in group}\;i. \end{array}\right.$$

We then have that the ANOVA model is a conditional normal distribution *p*( *y*∣**x**) that is of the same form as that used by Olkin and Tate (1961) to express correlations between discrete and continuous variables (see also Tate, 1954, 1966). To complete their model, Olkin and Tate (1961) specified a categorical (or multinomial) distribution for the indicator variables, that is,

(5) $$p(x)=\underset{i}{\prod }\pi_{i}^{x_{i}},$$

where *π*_*i*_ expresses the probability that a subject is assigned to a group *i*. With this multinomial distribution, we are able to cover many designs. In the derivation of *ω* ^2^, Hays (1963) assumed that the probabilities *π*_*i*_ are uniform for every cell in the design, and expressions for *η* ^2^ typically generalize this assumption to incorporate unbalanced designs. To foreshadow our later results, note that posterior inference on the parameters of the ANOVA model will be unaffected by a particular choice of model *p*(**x**) for the design/indicator variables whenever the design model *p*(**x**) does not depend on the parameters of the ANOVA model.

Before we analyze four common ANOVA designs, we first define two correlation measures that are closely related to the squared multiple correlation: the semipartial correlation coefficient (also known as the part correlation coefficient) and the partial correlation coefficient. Suppose that we wish to express the contribution of some subset **x**_*A*_ of the design variables **x** = {**x**_*A*_,**x**_*B*_} (i.e., $x_{A}\in D_{A}$, $x_{B}\in D_{B}$, and $D=D_{A}\times D_{B}$). The squared semipartial correlation $\rho_{y\;x_{A}|x_{B}}^{2}$ is then defined as (Cohen, 1982, p. 308)

(6) $$\rho_{y\;x_{A}|x_{B}}^{2}=\rho_{y\;x}^{2}-\rho_{y\;x_{B}}^{2}=\frac{\Delta^{\text{T}}\Gamma^{-1}\Delta -\Delta_{B}^{T}\Gamma_{B}^{-1}\Delta_{B}}{\Psi },$$

where $\rho_{y\;x_{B}}^{2}$ is the squared multiple correlation coefficient between *y* and the (sub)set **x**_*B*_ (i.e., the correlation obtained by removing any columns and rows in **Σ** that correspond to the set **x**_*A*_). The squared semipartial correlation $\rho_{y\;x_{A}|x_{B}}^{2}$ expresses the proportion of the variance in the outcome *y* that can be uniquely attributed to the subset **x**_*A*_. It is the population value of the *R* ^2^‐change statistic that is commonly used in multiple linear regression. Consequently, the squared partial correlation coefficient is defined as (Cohen, 1982, p. 308)

(7) $$\rho_{y\;x_{A}\cdot x_{B}}^{2}=\frac{\rho_{y\;x_{A}|x_{B}}^{2}}{1-\rho_{y\;x_{B}}^{2}}=\frac{\Delta^{T}\Gamma^{-1}\Delta -\Delta_{B}^{T}\Gamma_{B}^{-1}\Delta_{B}}{\Psi -\Delta_{B}^{T}\Gamma_{B}^{-1}\Delta_{B}},$$

where $1-\rho_{y\;x_{B}}^{2}$ denotes the proportion of variance in *y* that is not explained by **x**_*B*_. From this expression, we see that the squared semipartial correlation $\rho_{y\;x_{A}|x_{B}}^{2}$ expresses the proportion of the variance that can be attributed to the subset **x**_*A*_ after the unique contribution of **x**_*B*_ has been completely removed.

## 3 ANALYSIS I: AN ANOVA DESIGN WITH ONE FIXED FACTOR

Consider an ANOVA design with a single fixed factor consisting of *n* levels, that is,

$$y=\beta_{0}+\overset{n}{\underset{i=1}{\sum }}\beta_{i}x_{i}+\epsilon \text{,}\;\epsilon ∼N(0,\;\sigma^{2}),$$

where *x*_*i*_ is an indicator variable, with

$$x_{i}=\left\{\begin{array}{cc} 1,\; & \text{if subject is in group}\;i\\ 0,\; & \text{if subject is not in group}\;i \end{array}\right.$$

such that *β*_0_ + *β*_*i*_ is the expectation of *y* in group *i*, and it is assumed that the (residual) variance *σ* ^2^ is constant within each group. For completeness, we note here that the parameters in the conditional model *p*( *y*∣**x**) are not identified due to the use of dummy variables for the conditions. To identify the model parameters, we impose the sum‐to‐zero constraint *β*_1_ + ⋯ + *β*_*n*_ = 0. Upon assuming the multinomial distribution in Equation (5) for the indicator variables, it is then easy to confirm that we obtain the matrix **Σ** with the following elements:

$$\begin{array}{ll} \Psi & =\mathrm{Var}(y)=\sigma^{2}+\overset{n}{\underset{i=1}{\sum }}\pi_{i}{\left(\beta_{i}-\beta \right)}^{2}\\ \Gamma_{ij} & =\text{Cov}(x_{i},\;x_{j})=\left\{\begin{array}{cc} \pi_{i}(1-\pi_{i}),\; & \text{if}\;\;i=j\\ -\pi_{i}\pi_{j},\; & \text{if}\;\;i\neq j \end{array}\right.\\ \Delta_{i} & =\text{Cov}(y,\;x_{i})=\pi_{i}(\beta_{i}-\beta ), \end{array}$$

where we have used *β* to denote the weighted average $\sum_{i}\pi_{i}\beta_{i}$.

For the definition of the squared multiple correlation coefficient in Equation (2), we require the inverse of

(8) $$\Gamma =\mathrm{Var}(x)=\text{diag}(𝛑)-𝛑{𝛑}^{T},$$

where $𝛑={\left(\pi_{1},\;\pi_{2},\;\text{…}\;,\;\pi_{n}\right)}^{T}$, which is noninvertible due to the constraint $\sum_{i}x_{i}=1$. Without loss of generality, however, we can omit the contribution of one of the *x*_*i*_'s (c.f. Olkin & Tate, 1960). Let $\tilde{\Gamma }$ denote the matrix **Γ** with the *i*th row and column removed, and let $\tilde{\Delta }$ denote the vector **Δ** with the *i* entry removed. In the Appendix, we omit the contribution of *x*_*n*_ and show that

$${\tilde{\Delta }}^{T}{\tilde{\Gamma }}^{-1}\tilde{\Delta }=\overset{n}{\underset{i=1}{\sum }}\pi_{i}{\left(\beta_{i}-\beta \right)}^{2}.$$

Consequently, the expression for the squared multiple correlation coefficient is

(9) $$\rho_{y\;x}^{2}=\frac{\sum_{i=1}^{n}\pi_{i}{\left(\beta_{i}-\beta \right)}^{2}}{\sigma^{2}+\sum_{i=1}^{n}\pi_{i}{\left(\beta_{i}-\beta \right)}^{2}},$$

which is equation (3.12) from Olkin and Tate (1961).

In Equation (9), we see that the multinomial probabilities weigh the individual contributions from each group. In a balanced design, we assume that the weights (probabilities) are the same for each group (i.e., *π*_*i*_ = *n* ^−1^), and in that case, Equation (9) simplifies to

$$\rho_{y\;x}^{2}=\frac{\frac{1}{n}\sum_{i=1}^{n}\beta_{i}^{2}}{\sigma^{2}+\frac{1}{n}\sum_{i=1}^{n}\beta_{i}^{2}},$$

due to the sum‐to‐zero constraint; $\sum_{i}\pi_{i}\beta_{i}=n^{-1}\sum_{i}\beta_{i}=0$. This is the expression for *ω* ^2^ given by Hays (1963, p. 382). Furthermore, replacing the cell probabilities with observed cell proportions gives *η* ^2^. Gelman and Pardoe (2006) replace $\sum_{i}\pi_{i}{\left(\beta_{i}-\beta \right)}^{2}$ with the posterior expectation of $\frac{1}{n-1}\sum_{i}{\left(\beta_{i}-\bar{\beta }\right)}^{2}=\frac{1}{n-1}\sum_{i}\beta_{i}^{2}$, that is, the posterior expectation of the sample variance of the category parameters *β*_*i*_, which does not conform to an expression based on a multinomial distribution as the cell weights do not add to one. As a result, the factor contribution can be overestimated for designs with factors that have a small number of levels.

## 4 ANALYSIS II: AN ANOVA DESIGN WITH TWO FIXED FACTORS

In this section, we analyze an ANOVA design with two fixed factors. First, we consider a model with the two main effects but without the interaction, as this model is a direct extension of the single fixed factor discussed in the previous section. Second, we investigate the complete, full‐factorial model that includes both the two main effects as well as the interaction.

### 4.1 Two main effects model

Consider an ANOVA model with two fixed factors consisting of *n*_1_ and *n*_2_ levels, respectively, and no interaction effects, that is,

$$y=\beta_{0}+\overset{n_{1}}{\underset{i=1}{\sum }}\beta_{1i}x_{1i}+\overset{n_{2}}{\underset{j=1}{\sum }}\beta_{2j}x_{2j}+\epsilon \text{,}\;\epsilon ∼N(0,\;\sigma^{2}),$$

where *β*_0_ + *β*_1*i*_ + *β*_2*j*_ is the expectation of *y* in cell (*i*,*j*) of the *n*_1_ × *n*_2_ design matrix, and *x*_*f* *i*_ are indicator variables, with

$$x_{fi}=\left\{\begin{array}{cc} 1,\; & \text{if subject is in group}\;i\;\text{of factor}\;f\\ 0,\; & \text{if subject is not in group}\;i\;\text{of factor}\;f, \end{array}\right.$$

and it is assumed that the (residual) variance *σ* ^2^ is constant across all cells. Upon assuming independent multinomial distributions for the indicator variables of each factor (i.e., assuming that the manipulated factors are orthogonal), we obtain the matrix **Σ** with the following elements:

$$\begin{array}{ll} \Psi & =\mathrm{Var}(y)=\sigma^{2}+\overset{2}{\underset{f=1}{\sum }}\overset{n_{f}}{\underset{i=1}{\sum }}\pi_{fi}{\left(\beta_{fi}-\beta_{f}\right)}^{2}\\ \Gamma_{ij} & =\text{Cov}(x_{fi},\;x_{f^{\prime }j})=\left\{\begin{array}{cc} \pi_{fi}(1-\pi_{fi}),\; & \text{if}\;i=j\;\text{and}\;f=f^{\prime }\\ -\pi_{fi}\pi_{fj},\; & \text{if}\;i\neq j\;\text{and}\;f=f^{\prime }\\ 0,\; & \text{if}\;f\neq f^{\prime } \end{array}\right.\\ \Delta_{fi} & =\text{Cov}(y,\;x_{fi})=\pi_{fi}(\beta_{fi}-\beta_{f}), \end{array}$$

where we have used *β*_*f*_ to denote the weighted average $\sum_{i}\pi_{fi}\beta_{fi}$.

To compute the squared multiple correlation coefficient using (2), we require the inverse of

$$\Gamma =\left(\begin{array}{cc} \text{Cov}(x_{1},\;x_{1}) & \text{Cov}(x_{1},\;x_{2})\\ \text{Cov}(x_{2},\;x_{1}) & \text{Cov}(x_{2},\;x_{2}) \end{array}\right)=\left(\begin{array}{cc} \Gamma_{1} & 0\\ 0 & \Gamma_{2} \end{array}\right),$$

where **Γ**_*f*_ is the covariance matrix of the indicator variables for factor *f* as defined in Equation (8). Since **Γ** is a block‐diagonal matrix, we only require $\Gamma_{1}^{-1}$ and $\Gamma_{2}^{-1}$ to express **Γ** ^−1^. Using the results from the Appendix, where ${\tilde{\Gamma }}_{f}$ denotes the matrix **Γ**_*f*_ with the final column and row removed, for *f* = 1,2, we obtain

$$\rho_{y\;x}^{2}=\frac{\sum_{f=1}^{2}\sum_{i=1}^{n_{f}}\pi_{fi}{\left(\beta_{fi}-\beta_{f}\right)}^{2}}{\sigma^{2}+\sum_{f=1}^{2}\sum_{i=1}^{n_{f}}\pi_{fi}{\left(\beta_{fi}-\beta_{f}\right)}^{2}},$$

which is similar to the single‐fixed‐factor case that we have analyzed earlier.

We will now consider the semipartial and partial correlation for the unique contribution of the first factor after the contribution of the second factor has been partialed out. From the definition in Equation (6), we readily find the semipartial correlation

$$\rho_{y\;x_{1}|x_{2}}^{2}=\frac{\sum_{i=1}^{n_{1}}\pi_{1i}{\left(\beta_{1i}-\beta_{1}\right)}^{2}}{\sigma^{2}+\sum_{f=1}^{2}\sum_{i=1}^{n_{f}}\pi_{fi}{\left(\beta_{fi}-\beta_{f}\right)}^{2}},$$

and we can confirm that $\rho_{y\;x}^{2}=\rho_{y\;x_{1}|x_{2}}^{2}+\rho_{y\;x_{2}|x_{1}}^{2}$. Similarly, we can find the partial correlation

$$\rho_{y\;x_{1}\cdot x_{2}}^{2}=\frac{\sum_{i=1}^{n_{1}}\pi_{1i}{\left(\beta_{1i}-\beta_{1}\right)}^{2}}{\sigma^{2}+\sum_{i=1}^{n_{1}}\pi_{1i}{\left(\beta_{1i}-\beta_{1}\right)}^{2}},$$

which is identical to the expression for the multiple squared correlation in a single‐fixed‐factor design.

### 4.2 Full‐factorial model

Next, consider the ANOVA model with both main effects and the interaction, that is,

$$y=\beta_{0}+\overset{n_{1}}{\underset{i=1}{\sum }}\beta_{1i}x_{1i}+\overset{n_{2}}{\underset{j=1}{\sum }}\beta_{2j}x_{2j}+\overset{n_{1}}{\underset{i=1}{\sum }}\overset{n_{2}}{\underset{j=1}{\sum }}\beta_{3ij}x_{3ij}+\epsilon \text{,}\;\epsilon ∼N(0,\;\sigma^{2}),$$

where *β*_0_ + *β*_1*i*_ + *β*_2 *j*_ + *β*_3*i* *j*_ is the expectation of *y* in a cell (*i*,*j*) of the *n*_1_ × *n*_2_ design matrix, and *x*_3*i* *j*_ are indicator variables, with

$$x_{3ij}=x_{1i}\times x_{2j}=\left\{\begin{array}{cc} 1,\; & \text{if subject is in cell}\;(i,\;j)\\ 0,\; & \text{if subject is not in cell}\;(i,\;j). \end{array}\right.$$

To identify the model, we again adopt the sum‐to‐zero constraint as follows:

$$\overset{n_{1}}{\underset{i=1}{\sum }}\beta_{1i}=\overset{n_{2}}{\underset{j=1}{\sum }}\beta_{2j}=\overset{n_{1}}{\underset{i=1}{\sum }}\beta_{3ij}=\overset{n_{2}}{\underset{j=1}{\sum }}\beta_{3ij}=0.$$

By assuming that the indicator variables encoding the main effects have independent multinomial distributions for each factor, we find that the indicator variables encoding the interaction effects also have a multinomial distribution, that is,

(10) $$p(x_{3})=\overset{n_{1}}{\underset{i=1}{\prod }}\overset{n_{2}}{\underset{j=1}{\prod }}{\left(\pi_{1i}\times \pi_{2j}\right)}^{x_{3ij}}=\overset{n_{1}\times n_{2}}{\underset{k=1}{\prod }}\pi_{3k}^{x_{3k}},$$

where the latter provides shorthand notation that is useful later on.

With the two‐main‐effects‐only model, we could use the results from the Appendix and omit a row and column in **Γ** and **Δ** for each of the main effects, that is, rows and columns that correspond to $x_{1n_{1}}$ and $x_{2n_{2}}$, to express ${\tilde{\Delta }}^{T}{\tilde{\Gamma }}^{-1}\tilde{\Delta }$. This is not the case for the full‐factorial design, however, since even after omitting the rows corresponding to $x_{1n_{1}}$ and $x_{2n_{2}}$ (and $x_{3n_{3}}$), the inverse ${\tilde{\Gamma }}^{-1}$ does not exist. We can think of two possible solutions to this problem. The first is to remove the contributions of $x_{1n_{1}}$ and $x_{2n_{2}}$ altogether, that is, remove a single row and column from both **Γ**_1_ and **Γ**_2_, and remove *n*_1_ + *n*_2_ − 1 rows and columns from **Γ**_3_. However, as a result of leaving out these elements from **Γ**, and thus **Δ**, we lose much information about the design. We therefore take a different approach and use the main effects {**x**_1_,**x**_2_} in isolation from **x**_3_ to express ${\tilde{\Delta }}^{T}{\tilde{\Gamma }}^{-1}\tilde{\Delta }$, and vice versa. We will see that both the main effects and the interaction effects can be obtained with this approach.

First, we will study the model implied by only using the indicator variables encoding the interaction **x**_3_ and ignore the {**x**_1_,**x**_2_} variables. That is, we will first use **Γ**=Var(**x**_3_) and **Δ**=Cov(**x**_3_,*y*) for **Σ**. Second, we will ignore **x**_3_ and study the contribution of the main effects using **Γ**=Var({**x**_1_,**x**_2_}) and **Δ**=Cov({**x**_1_,**x**_2_},*y*) for **Σ**. Here, we can isolate the unique contribution of the main effects. By combining the two approaches, we can subsequently isolate the unique contributions of main effects and interaction and express these contributions in the form of semipartial and partial correlations. Thus, the solution comprises several steps that will be detailed below.

In the first step, we consider the use of **x**_3_ encoding the interaction and ignore the {**x**_1_,**x**_2_} variables. It is easy to confirm that for this situation, **Σ** has the elements

$$\begin{array}{ll} \Psi & =\mathrm{Var}(y)=\sigma^{2}+\overset{n_{1}}{\underset{i=1}{\sum }}\overset{n_{2}}{\underset{j=1}{\sum }}\pi_{1i}\pi_{2j}{\left(\beta_{ij}-\beta \right)}^{2}\\ \Gamma_{kl} & =\text{Cov}(x_{3k},\;x_{3l})=\left\{\begin{array}{cc} \pi_{3k}(1-\pi_{3k}),\; & \text{if}\;k=l\\ -\pi_{3k}\pi_{3l},\; & \text{if}\;k\neq l \end{array}\right.\\ \Delta_{k} & =\text{Cov}(y,\;x_{3k})=\text{Cov}(y,\;x_{3ij})=\pi_{1i}\pi_{2j}(\beta_{ij}-\beta ), \end{array}$$

where we have used the shorthand notation from Equation (10), that is, *β*_*i* *j*_ to denote *β*_1*i*_ + *β*_2 *j*_ + *β*_3*i* *j*_, *β* to denote *β*_1_ + *β*_2_ + *β*_3_, and *β*_3_ to denote the weighted average $\sum_{ij}\pi_{ij}\beta_{3ij}$. Since **Γ** is the covariance matrix of a single multinomial random variable, we have that (e.g., omitting *x*_*n*_ as in the Appendix)

$${\tilde{\Delta }}^{T}{\tilde{\Gamma }}^{-1}\tilde{\Delta }=\overset{n_{1}}{\underset{i=1}{\sum }}\overset{n_{2}}{\underset{j=1}{\sum }}\pi_{1i}\pi_{2j}{\left(\beta_{1i}+\beta_{2j}+\beta_{3ij}-\beta \right)}^{2}.$$

Thus, the squared multiple correlation coefficient can be expressed as

$$\rho_{y\;x}^{2}=\frac{\sum_{i=1}^{n_{1}}\sum_{j=1}^{n_{2}}\pi_{1i}\pi_{2j}{\left(\beta_{1i}+\beta_{2j}+\beta_{3ij}-\beta \right)}^{2}}{\sigma^{2}+\sum_{i=1}^{n_{1}}\sum_{j=1}^{n_{2}}\pi_{1i}\pi_{2j}{\left(\beta_{1i}+\beta_{2j}+\beta_{3ij}-\beta \right)}^{2}},$$

which, however, does not partition into separate terms for the main effects and the interaction when the cell probabilities *π*_*i* *j*_ are not uniform. When the cell probabilities are uniform, that is, $\pi_{ij}=\frac{1}{n_{1}\times n_{2}}$, the squared multiple correlation coefficient equals

(11) $$\rho_{y\;x}^{2}=\frac{\frac{\sum_{i=1}^{n_{1}}\beta_{1i}^{2}}{n_{1}}+\frac{\sum_{j=1}^{n_{2}}\beta_{2j}^{2}}{n_{2}}+\frac{\sum_{i=1}^{n_{1}}\sum_{j=1}^{n_{2}}\beta_{3ij}^{2}}{n_{1}\times n_{2}}}{\sigma^{2}+\frac{\sum_{i=1}^{n_{1}}\beta_{1i}^{2}}{n_{1}}+\frac{\sum_{j=1}^{n_{2}}\beta_{2j}^{2}}{n_{2}}+\frac{\sum_{i=1}^{n_{1}}\sum_{j=1}^{n_{2}}\beta_{3ij}^{2}}{n_{1}\times n_{2}}},$$

which does partition into separate terms for the main effects and the interaction. However, that the total sum of squares partitions in this manner is insufficient to partial out an effect. We therefore now turn to the model implied by {**x**_1_,**x**_2_}.

In the second step, we consider the covariance matrix that is implied by ignoring the indicator variables encoding the interaction and only consider the indicator variables for the main effects, that is, {**x**_1_,**x**_2_}. It is easy to confirm that, for this situation, the covariance matrix **Σ** has the elements

$$\begin{array}{ll} \Psi & =\mathrm{Var}(y)=\sigma^{2}+\overset{n_{1}}{\underset{i=1}{\sum }}\overset{n_{2}}{\underset{j=1}{\sum }}\pi_{3ij}{\left(\beta_{ij}-\beta \right)}^{2}\\ \Gamma_{ij} & =\text{Cov}(x_{fi},\;x_{f^{\prime }j})=\left\{\begin{array}{cc} \pi_{fi}(1-\pi_{fi}),\; & \text{if}\;i=j\;\text{and}\;f=f^{\prime }\\ -\pi_{fi}\pi_{fj},\; & \text{if}\;i\neq j\;\text{and}\;f=f^{\prime }\\ 0,\; & \text{if}\;f\neq f^{\prime } \end{array}\right.\\ \Delta_{1i} & =\text{Cov}(y,\;x_{1i})=\pi_{1i}\left(\beta_{1i}-\beta_{1}+\underset{j}{\sum }\pi_{2j}\beta_{3ij}-\beta_{3}\right)\\ \Delta_{2i} & =\text{Cov}(y,\;x_{2i})=\pi_{2i}\left(\beta_{2i}-\beta_{2}+\underset{i}{\sum }\pi_{1i}\beta_{3ij}-\beta_{3}\right), \end{array}$$

where the covariance matrix **Γ** is identical to the covariance matrix for the two main effects analysis that we considered before. As a result, we find the squared multiple correlation coefficient, as follows:

$$\begin{array}{ll} \rho_{y\;\{x_{1},\;x_{2}\}}^{2} & =\frac{1}{\Psi }\left(\overset{n_{1}}{\underset{i=1}{\sum }}\pi_{1i}{\left(\beta_{1i}-\beta_{1}+\underset{j}{\sum }\pi_{2j}\beta_{3ij}-\beta_{3}\right)}^{2}\right.\\ & \;+\left.\overset{n_{2}}{\underset{j=1}{\sum }}\pi_{2j}{\left(\beta_{2j}-\beta_{2}+\underset{i}{\sum }\pi_{1i}\beta_{3ij}-\beta_{3}\right)}^{2}\right), \end{array}$$

which does not simplify further in the case of nonuniform cell probabilities ***π***. Assuming uniform cell probabilities, that is, $\pi_{1i}=n_{1}^{-1}$ and $\pi_{2j}=n_{2}^{-1}$, we observe that this expression of the squared multiple correlation simplifies to

$$\begin{array}{ll} \rho_{y\;\{x_{1},\;x_{2}\}}^{2} & =\frac{\frac{\sum_{i=1}^{n_{1}}\beta_{1i}^{2}}{n_{1}}+\frac{\sum_{j=1}^{n_{2}}\beta_{2j}^{2}}{n_{2}}}{\sigma^{2}+\frac{\sum_{i=1}^{n_{1}}\beta_{1i}^{2}}{n_{1}}+\frac{\sum_{j=1}^{n_{2}}\beta_{2j}^{2}}{n_{2}}+\frac{\sum_{i=1}^{n_{1}}\sum_{j=1}^{n_{2}}\beta_{3ij}^{2}}{n_{1}\times n_{2}}}, \end{array}$$

from which one can also recognize a semipartial correlation, as the contribution of the interaction effect has been removed from the numerator but not from the denominator.

We can now consider the semipartial and partial correlations that express the contribution of the first main effect. Since we have seen that $\rho_{y\;\{x_{1},\;x_{2}\}}^{2}=\rho_{y\;\{x_{1},\;x_{2}\}|x_{3}}^{2}$, we have

$$\begin{array}{ll} \rho_{y\;x_{1}|x_{2}}^{2}=\rho_{y\;\{x_{1},\;x_{2}\}}^{2}-\rho_{y\;x_{2}}^{2}=\rho_{y\;\{x_{1},\;x_{2}\}|x_{3}}^{2}-\rho_{y\;x_{2}|x_{3}}^{2}=\rho_{y\;x_{1}|\{x_{2},\;x_{3}\}}^{2}. & \end{array}$$

As a result, we find the following expression for the squared semipartial correlation for the first main effect:

$$\rho_{y\;x_{1}|\{x_{2},\;x_{3}\}}^{2}=\frac{1}{\Psi }\overset{n_{1}}{\underset{i=1}{\sum }}\pi_{1i}{\left(\beta_{1i}-\beta_{1}+\underset{j}{\sum }\pi_{2j}\beta_{3ij}-\beta_{3}\right)}^{2}.$$

Note that when the cell probabilities are assumed to be uniform, this expression simplifies to

$$\rho_{y\;x_{1}|\{x_{2},\;x_{3}\}}^{2}=\frac{\frac{\sum_{i=1}^{n_{1}}\beta_{1i}^{2}}{n_{1}}}{\sigma^{2}+\frac{\sum_{i=1}^{n_{1}}\beta_{1i}^{2}}{n_{1}}+\frac{\sum_{j=1}^{n_{2}}\beta_{2j}^{2}}{n_{2}}+\frac{\sum_{i=1}^{n_{1}}\sum_{j=1}^{n_{2}}\beta_{ij}^{2}}{n_{1}\times n_{2}}}.$$

From here, we can find the squared partial correlation as

$$\rho_{y\;x_{1}\cdot \{x_{2},\;x_{3}\}}^{2}=\frac{\frac{\sum_{i=1}^{n_{1}}\beta_{1i}^{2}}{n_{1}}}{\sigma^{2}+\frac{\sum_{i=1}^{n_{1}}\beta_{1i}^{2}}{n_{1}}},$$

which is again simply the expression for the multiple squared correlation in a single‐fixed‐factor design.

We have shown how to express the semipartial and partial correlation (ES) measures that correspond to an ANOVA design with two fixed factors. In particular, we have seen simple expressions for the case with uniform cell probabilities, since, in that case, the total sum of squares $\sigma_{D}^{2}$ decomposes into distinct terms for the main effects and the interaction (c.f. Equation (11)), that is,

$$\sigma_{D}^{2}=\sigma_{A}^{2}+\sigma_{B}^{2}+\sigma_{A\times B}^{2},$$

with the terms denoting the contribution of the main effect from a factor A, factor B, and the interaction between A and B, respectively. As a result, we see that the semipartial correlation $\rho_{y\;x_{1}|\{x_{2},\;x_{3}\}}^{2}$ expresses the unique contribution of the first factor, that is,

$$\frac{\sigma_{A}^{2}}{\sigma_{E}^{2}+\sigma_{A}^{2}+\sigma_{B}^{2}+\sigma_{A\times B}^{2}}.$$

For a long time, this expression was proposed as a “partial‐*ω* ^2^”/“partial‐*η* ^2^” in the extension of *ω* ^2^ and *η* ^2^ measures for single‐factor designs (Kerlinger, 1964; Kennedy, 1970) and consequently used by statistical software packages such as SPSS (Levine & Hullet, 2002; Pierce et al., 2004). Others suggested a different expression (e.g., Cohen, 1968, 1973; Keren & Lewis, 1979), that is,

(12) $$\frac{\sigma_{A}^{2}}{\sigma_{E}^{2}+\sigma_{A}^{2}},$$

since it “can be shown that in the same way ω^2^ is a squared multiple correlation (12) is a squared partial correlation, with the artificial predictor variables designating B and the AB interaction partialled out” (Keren & Lewis, 1979, p. 123). However, this is true only for the particular case of uniform cell probabilities (e.g., *ω* ^2^), since, for the nonuniform case (e.g., *η* ^2^), the partial correlation is found to be

$$\frac{\sum_{i}\pi_{1i}{\left(\beta_{1i}-\beta_{1}+\sum_{j}\pi_{2j}\beta_{3ij}-\beta_{3}\right)}^{2}}{\sigma^{2}+\sum_{i}\pi_{1i}{\left(\beta_{1i}-\beta_{1}+\sum_{j}\pi_{2j}\beta_{3ij}-\beta_{3}\right)}^{2}},$$

and it is clear that we cannot completely partial out the interaction. That is, the (semi)partial measures do not express unique contributions in the nonuniform case.

## 5 ANALYSIS III: AN ANOVA DESIGN WITH ONE FIXED FACTOR AND ONE COVARIATE

Consider an ANOVA design with a single fixed factor consisting of *n* levels and a (set of *m*) covariate(s), that is,

$$y=\beta_{0}+\overset{n}{\underset{i=1}{\sum }}\beta_{1i}x_{1i}+\overset{m}{\underset{j=1}{\sum }}\beta_{2j}x_{2j}+\epsilon ,\;\epsilon ∼N(0,\;\sigma^{2}),$$

where $\beta_{0}+\beta_{1i}+\sum_{j=1}^{m}\beta_{2j}x_{2j}$ is the expectation of *y* for a subject in group *i* with covariate (vector) **x**_2_. As before, we assume that the indicator variables are multinomial random variables and that the distribution of the indicator variables is independent of that of the covariates, that is, *p*(**x**_1_,**x**_2_) = *p*(**x**_1_)*p*(**x**_2_). For the specification of the covariance matrix, we have to specify the covariance matrix **Γ**_2_ = Var(**x**_2_), which depends on the choice of *p*(**x**_2_). The work of Olkin and Tate (1961) focused on the case where *p*(**x**_2_) is a multivariate normal that depends on **x**_1_.

We consider here the case of a single covariate, which allows us to provide an analytic expression for the squared correlation coefficient in the general case requiring only that we are able to specify Var(*x*_2_), since it is easy to confirm that the covariance matrix **Σ** has the elements

$$\begin{array}{ll} \Psi & =\mathrm{Var}(y)=\sigma^{2}+\overset{n_{1}}{\underset{i=1}{\sum }}\pi_{1i}{\left(\beta_{1i}-\beta_{1}\right)}^{2}+\beta_{2}^{2}\mathrm{Var}(x_{2})\\ \Gamma_{1ij} & =\text{Cov}(x_{1i},\;x_{1j})=\left\{\begin{array}{cc} \pi_{1i}(1-\pi_{1i}),\; & \text{if}\;i=j\\ -\pi_{1i}\pi_{1j},\; & \text{if}\;i\neq j \end{array}\right.\\ \Gamma_{12\;i\cdot } & =\text{Cov}(x_{1i},\;x_{2})=0\\ \Gamma_{2} & =\mathrm{Var}(x_{2})\\ \Delta_{1i} & =\text{Cov}(y,\;x_{1i})=\pi_{1i}(\beta_{1i}-\beta_{1})\\ \Delta_{2} & =\text{Cov}(y,\;x_{2})=\beta_{2}\mathrm{Var}(x_{2}). \end{array}$$

If one is willing to assume that the distribution of the covariate is normal with mean *μ* and variance *τ* ^2^, for instance, then we may replace Var(*x*_2_) with *τ* ^2^. Similarly, one could use the observed variance.

By assuming that the indicator variables and the covariate(s) are independent, we observe that the covariance matrix **Γ** is (block‐)diagonal, such that we can apply the results from the Appendix to obtain

$$\rho_{y\;\{x_{1},\;x_{2}\}}=\frac{\sum_{i=1}^{n_{1}}\pi_{1i}{\left(\beta_{1i}-\beta_{1}\right)}^{2}+\beta_{2}^{2}\tau^{2}}{\sigma^{2}+\sum_{i=1}^{n_{1}}\pi_{1i}{\left(\beta_{1i}-\beta_{1}\right)}^{2}+\beta_{2}^{2}\tau^{2}}.$$

From here, it is simple to find the semipartial correlation for the covariate as

$$\rho_{y\;x_{2}|x_{1}}=\frac{\beta_{2}^{2}\tau^{2}}{\sigma^{2}+\sum_{i=1}^{n_{1}}\pi_{1i}{\left(\beta_{1i}-\beta_{1}\right)}^{2}+\beta_{2}^{2}\tau^{2}}$$

and, similarly, the partial correlation as

$$\rho_{y\;x_{2}\cdot x_{1}}=\frac{\beta_{2}^{2}\tau^{2}}{\sigma^{2}+\beta_{2}^{2}\tau^{2}}.$$

When more than one covariate is used, it is clear that the expressions above depend on the covariance structure **Γ**_2_. This is illustrated in the next analysis, where we consider a bivariate normal distribution for the random effects, which is one specific generalization of the results that we obtained here.

## 6 ANALYSIS IV: AN ANOVA DESIGN WITH ONE FIXED AND TWO CORRELATED RANDOM FACTORS

Consider an ANOVA design with a single fixed factor of *n* levels and a (set of *m*) random effect(s), that is,

$$y=\beta_{0}+\overset{n}{\underset{i=1}{\sum }}\beta_{1i}x_{1i}+\overset{m}{\underset{j=1}{\sum }}x_{2j}+\epsilon ,\;\epsilon ∼N(0,\;\sigma^{2}),$$

where $\beta_{0}+\beta_{1i}+\sum_{j=1}^{m}x_{2j}$ is the mean (expected) value of *y* for a subject in group *i* with random effect (values) **x**_2_. In typical applications, it is assumed that the random effects have a (multivariate) normal distribution with a zero‐mean vector (for identification) and covariance matrix **Λ**. We consider here the situation for two random effects.

We assume that the distribution of the indicator variables for the manipulated factor (**x**_1_) is a multinomial distribution (independent from the random effects) and that the distribution of the random effects is (bivariate) normal, with covariance matrix

$$\Gamma_{2}=\left(\begin{array}{cc} \tau_{1}^{2} & \varrho \tau_{1}\tau_{2}\\ \varrho \tau_{1}\tau_{2} & \tau_{2}^{2} \end{array}\right),$$

where *ϱ* denotes the correlation between the two random effects. It is easy to confirm that the covariance matrix **Σ** has the elements

$$\begin{array}{ll} \Psi & =\sigma^{2}+\overset{n_{1}}{\underset{i=1}{\sum }}\pi_{1i}{\left(\beta_{1i}-\beta_{1}\right)}^{2}+\tau_{1}^{2}+\tau_{2}^{2}+2\varrho \tau_{1}\tau_{2}\\ \Gamma_{1ij} & =\text{Cov}(x_{1i},\;x_{1j})=\left\{\begin{array}{cc} \pi_{1i}(1-\pi_{1i}),\; & \text{if}\;i=j\\ -\pi_{1i}\pi_{1j},\; & \text{if}\;i\neq j \end{array}\right.\\ \Gamma_{12\;ij} & =\text{Cov}(x_{1i},\;x_{2j})=0\\ \Delta_{1i} & =\text{Cov}(y,\;x_{1i})=\pi_{1i}(\beta_{1i}-\beta_{1})\\ \Delta_{2j} & =\text{Cov}(y,\;x_{2j})=\tau_{j}^{2}+\varrho \tau_{j}\tau_{3-j}. \end{array}$$

As before, we have assumed that the manipulated factors are independent of other variables, so that the covariance matrix **Γ** is block‐diagonal. Applying the results from the Appendix to **Γ**_1_ and from observing that

$$\Gamma_{2}^{-1}=\frac{1}{\tau_{1}^{2}\tau_{2}^{2}(1-\varrho^{2})}\left(\begin{array}{cc} \tau_{2}^{2} & -\varrho \tau_{1}\tau_{2}\\ -\varrho \tau_{1}\tau_{2} & \tau_{1}^{2} \end{array}\right),$$

we find the squared correlation coefficient as follows:

$$\rho_{y\;x}^{2}=\frac{\sum_{i=1}^{n_{1}}\pi_{i}{\left(\beta_{1i}-\beta_{1}\right)}^{2}+\tau_{1}^{2}+\tau_{2}^{2}+2\varrho \tau_{1}\tau_{2}}{\sigma^{2}+\sum_{i=1}^{n_{1}}\pi_{i}{\left(\beta_{1i}-\beta_{1}\right)}^{2}+\tau_{1}^{2}+\tau_{2}^{2}+2\varrho \tau_{1}\tau_{2}},$$

which explicitly uses the covariance between the random effects.

We can now consider semipartial and partial correlations for the first random effect. The semipartial correlation (i.e., removing the contribution of the fixed factor and the second random effect) describing the contribution of the first random effect is equal to

$$\rho_{y\;x_{21}|\{x_{1},\;x_{22}\}}^{2}=\frac{(1-\varrho^{2})\tau_{1}^{2}}{\sigma^{2}+\sum_{i=1}^{n_{1}}\pi_{i}{\left(\beta_{1i}-\beta_{1}\right)}^{2}+\tau_{1}^{2}+\tau_{2}^{2}+2\varrho \tau_{1}\tau_{2}},$$

which was to be expected, as $(1-\varrho^{2})\tau_{1}^{2}$ is the variance of *x*_21_ after conditioning on *x*_22_. That is, the partial correlation coefficient

$$\begin{array}{ll} \rho_{y\;x_{21}\cdot \{x_{1},\;x_{22}\}}^{2} & =\frac{(1-\varrho^{2})\tau_{1}^{2}}{\sigma^{2}+(1-\varrho^{2})\tau_{1}^{2}} \end{array}$$

is the intraclass correlation coefficient (Hays, 1963, p. 424).

## 7 BAYESIAN INFERENCE ON ρ ^2^

The correlation coefficients discussed in this paper are functions of the parameters from the correlation model

p(y,x∣β,σ,𝛌)=p(y|x,β,σ)p(x|𝛌),

where *p*( *y* | **x**,***β***,*σ*) denotes the ANOVA model that depends on cell means ***β*** and the residual variance *σ* ^2^, and *p*(**x** | ***λ***) is the distribution that we assume for the design variables and depends on the parameter vector ***λ*** (e.g., cell probabilities ***π*** and the population parameters of any random effects or covariates). By assigning prior probabilities *p*(***β***,*σ*) and *p*(***λ***) independently to the parameters {***β***,*σ*} and ***λ***, we observe that conditional upon the observed data **X** and **Y**, the parameters {***β***,*σ*} and ***λ*** are conditionally independent, that is,

p(β,σ,𝛌|X,Y)=p(β,σ|X,Y)p(𝛌|X).

This factorization confirms that posterior inference on {***β***,*σ*} is unaffected by a specific choice of model *p*(**x**|***λ***).

Posterior inference on the correlation coefficients proceeds by simulation: Generate ***β*** ^∗^ and *σ* ^∗^ as a sample from *p*(***β***,*σ* | **X**,**Y**) and ***λ*** ^∗^ as a sample from *p*(***λ*** | **X**). Given a simulated draw for each parameter of the joint model, that is, ***β*** ^∗^, *σ* ^∗^, and ***λ*** ^∗^, we can compute the correlation measure of interest. Repeating this procedure many times produces samples from the posterior of the correlation coefficient that we may use to produce Monte Carlo estimates of quantities of interest, such as posterior medians, means, credible intervals, etc. When the prior distributions *p*(***β***,*σ*) and *p*(***λ***) are proper probability distributions, one can evaluate the prior distribution on the correlation measure in a similar way: Generate ***β*** ^∗^, *σ* ^∗^, and ***λ*** ^∗^ from the prior distributions and use these values to compute the correlation measure as a sample from the prior.

Further elaboration of the design model and its parameter ***λ*** offers an interesting avenue for further study, for instance, in the case of targeted, unbalanced, or multistage experiments. However, in regular experimental analyses, we are usually not interested in ***λ*** (except perhaps in the case of random effects), and we can simply use the observed values (i.e., cell proportions, means, and covariances of the covariates). We therefore only require draws from the posterior distributions of the ANOVA model parameters {***β***,*σ*}. Procedures to sample from these posterior distributions are described in many publications (see, for instance, Gelman et al., 2014; Gelman, 2005) and oftentimes use Markov chain Monte Carlo methods. A convenient tool is the BayesFactor package that is available for GNU‐R and works for many of the commonly used ANOVA designs (Morey & Rouder, 2015).

### 7.1 Example I: Incorporating prior information in a single‐fixed‐factor design

To illustrate the use of our approach to $\rho_{y\;x}^{2}$, we consider data from an experiment performed by Gibson, Losee, and Vitiello (2014), which was part of a special issue “Replications of important results in social psychology” (Nosek & Lakens, 2013, 2014). In this experiment, Gibson et al. (2014) attempted to replicate the results of Shih, Pittinsky, and Ambady (1999) on *stereotype susceptibility*. Shih et al. (1999) had investigated the performance of Asian American women on a math test after the women had been surreptitiously attented to either their Asian identity (i.e., high maths ability stereotype), or their female identity (i.e., low maths ability stereotype), or have not been primed as such (control condition). The original results of Shih et al. (1999) suggested a relation between stereotype priming and maths performance: Subjects in the Asian identity condition outperformed subjects in the female identity and control conditions, and subjects in the control group outperformed subjects in the female identity condition.

In their replication attempt, Gibson et al. (2014) collected *N* = 158 participants: 52 in the Asian identity condition, 54 in the female identity condition, and 52 in the control condition. (The data for this example are available at https://osf.io/vnaqq/.) We analyzed the Gibson data as a single‐fixed‐factor design using a setup similar to that of Rouder, Morey, Speckman, and Province (2012), using noninformative Jeffreys priors on *β*_0_ and *σ* ^2^ and *g*‐priors for *β*_1_, *β*_2_, and *β*_3_ with the restriction that $\sum_{i}\beta_{i}=0$, where we use *β*_1_, *β*_2_, and *β*_3_ to refer to the departure of the overall mean for the Asian identity, female identity, and control conditions, respectively. For the squared correlation coefficient, we have used expression (9) and simply replaced the cell probabilities with the observed proportions.

In Table 1, we summarize the posterior distributions of the model parameters and the squared multiple correlation coefficient in terms of the posterior means, standard deviations, and the 2.5%, 50%, and 97.5% quantiles. Note that there is little variability in the mean departures between the three groups, indicating that the effect of the manipulation is small. This can also be observed from the posterior median of *ρ* ^2^ indicating that less than 2% of the total variance is attributed to the variability between conditions, with a small margin of uncertainty as indicated by the narrow 95% central credible interval that ranges from .001 to .062.

**Table 1:** Posterior summaries for model parameters and the squared multiple correlation for Example I

	Mean	SD	**2.5%**	**50%**	**95%**
*β*_0_	0.558	0.019	0.520	0.558	0.595
*β*_1_	0.029	0.025	−0.020	0.029	0.079
*β*_2_	−0.023	0.025	−0.072	−0.023	0.026
*β*_3_	−0.006	0.025	−0.055	−0.006	0.043
*σ* ^2^	0.057	0.007	0.046	0.057	0.071
$\rho_{y\;x}^{2}$	.019	.017	.001	.014	.062

Note. SD = standard deviation.

Note that Gibson et al. (2014) had available prior information from the original experiment of Shih et al. (1999). In particular, the original results suggested that the Asian identity group outperformed both the female identity and control groups and that the control group outperformed the female identity group. This information is easily incorporated into the prior distribution of the model parameters using the order constraint *β*_2_ < *β*_3_ < *β*_1_. It should be clear that when the prior constraint aligns with the observed data, we obtain an estimate of *ρ* ^2^ that is at least as high as the estimate of *ρ* ^2^ obtained without including prior constraints, but we would obtain a lower estimate when the prior constraint does not align with the observed data.

In Table 2, we show the posterior means, standard deviations, and the 2.5%, 50%, and 97.5% quantiles for the parameters and the multiple correlation using the prior constraint *β*_2_ < *β*_3_ < *β*_1_. From Table 2, we see that including the prior constraint increased the variability of the group mean discrepancies, whereas the error variability *σ* ^2^ remained the same. That is, *ρ* ^2^ increased. This was to be expected since the posterior results in Table 1 aligned with the hypothesis.

**Table 2:** Posterior summaries for model parameters and the squared multiple correlation for Example I using the prior restriction β_2_ < β_3_ < β_1_

	Mean	SD	**2.5%**	**50%**	**95%**
*β*_0_	0.558	0.019	0.521	0.558	0.595
*β*_1_	0.045	0.022	0.010	0.043	0.093
*β*_2_	−0.040	0.018	−0.080	−0.038	−0.010
*β*_3_	−0.006	0.015	−0.037	−0.005	0.024
*σ* ^2^	0.057	0.007	0.046	0.057	0.071
$\rho_{y\;x}^{2}$	.026	.021	.002	.022	.079

Note. SD = standard deviation.

**Figure 1:**
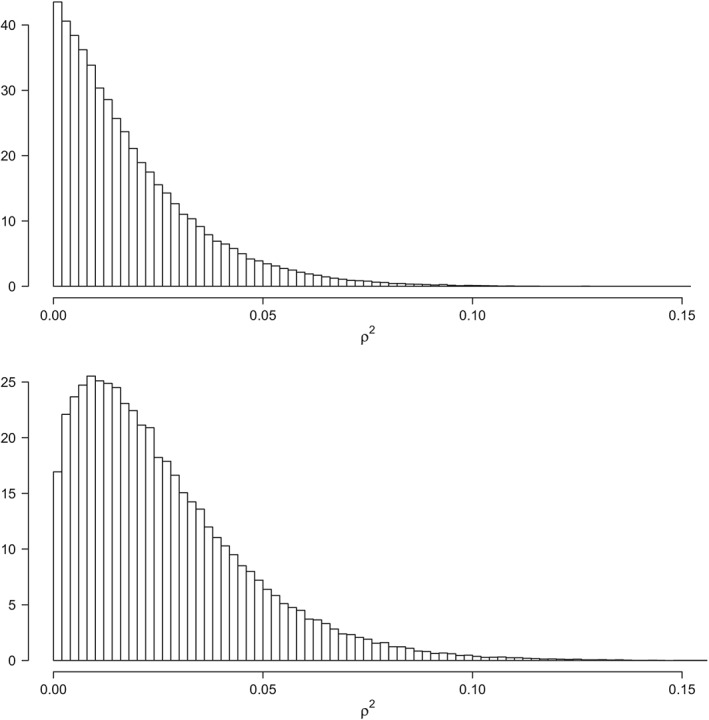
Histogram of 100,000 draws from the posterior distribution of ρ ^2^ given the unrestricted model in the top panel and the order‐restricted model in the bottom panel

An advantage of a Bayesian approach to interval estimation, as opposed to a classical approach, is that the intervals and related statistics are derived from the posterior distribution. In Figure 1, we show histograms of 100,000 posterior samples for *ρ* ^2^, with the top panel showing the posterior distribution of *ρ* ^2^ without the prior constraint and the bottom panel showing the posterior distribution with the prior constraint. Since the posterior is a probability distribution, we can ask questions such as: “What is the (posterior) probability that *ρ* ^2^ > 0.05?” From Figure 1, it should be obvious that this probability is smaller for the estimated posterior distribution shown in the top panel than in the estimated posterior distribution in the bottom panel. To wit, the probability that *ρ* ^2^ > .05 equaled approximately 5% for the estimated posterior distribution in the top panel and approximately 12% for the one shown in the bottom panel. Observe that with the improper Jeffreys prior on *σ* ^2^, the prior distribution on $\rho_{y\;x}^{2}$ is also improper. In fact, it will have all its mass placed at zero. This would mean that, in this case, the prior probability that $\rho_{y\;x}^{2}>0.05$ is zero.

### 7.2 Example II: An illustration of ρ ^2^‐change in a repeated‐measures design

To illustrate the use of the semipartial correlation coefficient as *ρ* ^2^‐change (Cohen, 1982), we consider data from Žeželj and Jokić (2014), which was also part of the *Social Psychology* special issue. (The data for this example are available at https://osf.io/z5te6/.) Žeželj and Jokić (2014) aimed at an exact replication of the results from Eyal, Liberman, and Trope (2008), who found that “people would judge immoral acts more harshly if presented to them as temporally distant rather than presented as temporally close” (Žeželj & Jokić, 2014, p. 225). The following experiment was conducted: Subjects were presented with three stories on morally offensive behaviors (eating one's dead pet, sexual intercourse with sibling, and dusting with the national flag) and were asked to imagine that the story would either happen the next day (near future) or the next year (distant future), after which participants were asked to judge the wrongness of these actions. Žeželj and Jokić (2014) collected data from *N* = 116 students, which were analyzed using a mixed‐ANOVA design with temporal distance and moral vignettes as between‐subject factors, that is, a 2 × 3 repeated‐measures design. We analyzed the data using the BayesFactor package (Morey & Rouder, 2015), using the main effects of temporal distance (fixed factor 1) and the moral vignettes (fixed factor 2), and a random participant factor.

Table 3 shows the posterior means, standard deviations, and the 2.5%, 50%, and 97.5% quantiles for the model parameters for this example. From Table 3, we see that there is little variability for the group mean deviations for the first factor—*β*_11_ and *β*_12_—but that there is nonnegligible variation in the mean deviations for the second factor—*β*_21_, *β*_22_, and *β*_23_—and the contribution of the effect—*τ* ^2^. We will study their contributions, in turn, using semipartial correlations.

**Table 3:** Posterior summaries for model parameters for Example II

	Mean	SD	**2.5%**	**50%**	**95%**
*β*_0_	−2.383	0.191	−2.759	−2.383	−2.007
*β*_11_	−0.056	0.181	−0.413	−0.055	0.299
*β*_12_	0.056	0.181	−0.299	0.055	0.413
*β*_21_	−1.535	0.164	−1.855	−1.535	−1.214
*β*_22_	−1.416	0.164	−1.736	−1.416	−1.092
*β*_23_	2.951	0.164	2.629	2.951	3.273
*σ* ^2^	4.688	0.437	3.907	4.663	5.613
*τ* ^2^	2.630	0.569	1.643	2.584	3.874

Note. SD = standard deviation.

For this example, the squared semipartial correlations are defined as

$$\begin{array}{ll} \rho_{y\;x_{1}|\{x_{2},\;x_{3}\}}^{2} & =\frac{\sum_{i=1}^{n_{1}}\pi_{1i}{\left(\beta_{1i}-\beta_{1}\right)}^{2}}{\sigma^{2}+\sum_{f=1}^{2}\sum_{i=1}^{n_{f}}\pi_{fi}{\left(\beta_{fi}-\beta_{f}\right)}^{2}+\tau^{2}}\\ \rho_{y\;x_{2}|\{x_{1},\;x_{3}\}}^{2} & =\frac{\sum_{i=1}^{n_{2}}\pi_{2i}{\left(\beta_{2i}-\beta_{2}\right)}^{2}}{\sigma^{2}+\sum_{f=1}^{2}\sum_{i=1}^{n_{f}}\pi_{fi}{\left(\beta_{fi}-\beta_{f}\right)}^{2}+\tau^{2}}\\ \rho_{y\;x_{3}|\{x_{1},\;x_{2}\}}^{2} & =\frac{\tau^{2}}{\sigma^{2}+\sum_{f=1}^{2}\sum_{i=1}^{n_{f}}\pi_{fi}{\left(\beta_{fi}-\beta_{f}\right)}^{2}+\tau^{2}}, \end{array}$$

where *τ* ^2^ is the variance of the random effect (i.e., subjects). The squared multiple correlation is defined as the sum of these three semipartial correlations, that is,

(13) $$\rho_{y\;x}^{2}=\rho_{y\;x_{1}|\{x_{2},\;x_{3}\}}^{2}+\rho_{y\;x_{2}|\{x_{1},\;x_{3}\}}^{2}+\rho_{y\;x_{3}|\{x_{1},\;x_{2}\}}^{2}.$$

From the posterior samples of the model parameters that we described in Table 3 and using the observed proportions ***π***_1_ = {.491,.509} and ${𝛑}_{2}=\left\{\frac{1}{3},\;\frac{1}{3},\;\frac{1}{3}\right\}$, we computed the squared correlation measures.

In Table 4, we summarize the posterior distributions of the squared semipartial correlations in terms of the posterior means, standard deviations, and the 2.5%, 50%, and 97.5% quantiles. As can be seen from Table 4 and was argued above, the amount of variation that can be attributed to the first fixed factor is very small, but the variation that can be uniquely attributed to the second fixed factor and the random factor is nonnegligible, with the posterior medians indicating that approximately 0.1%, 37.4%, and 22.2% of the total variance can be uniquely attributed to the first, second, and random factors, respectively, totaling approximately 60% of the variance being explained. While the 95% credible intervals for the semipartial correlations of the second factor (moral vignettes) and the random participant factor covered more than 10% of the total range, indicating that we are still uncertain about their exact values, the credible interval of the first factor (temporal distance) covered less than 2% of the total range, indicating here that we are fairly certain that temporal distance does not explain much variance.

**Table 4:** Posterior summaries for the squared semipartial and multiple correlations for Example II

	Mean	SD	**2.5%**	**50%**	**95%**
$\rho_{y\;x_{1}|\{x_{2},\;x_{3}\}}^{2}$	.003	.004	.000	.001	.015
$\rho_{y\;x_{2}|\{x_{1},\;x_{3}\}}^{2}$	.373	.033	.308	.374	.437
$\rho_{y\;x_{3}|\{x_{1},\;x_{2}\}}^{2}$	.223	.041	.147	.222	.307
$\rho_{y\;x}^{2}$	.600	.037	.524	.601	.667

Note. SD = standard deviation.

**Figure 2:**
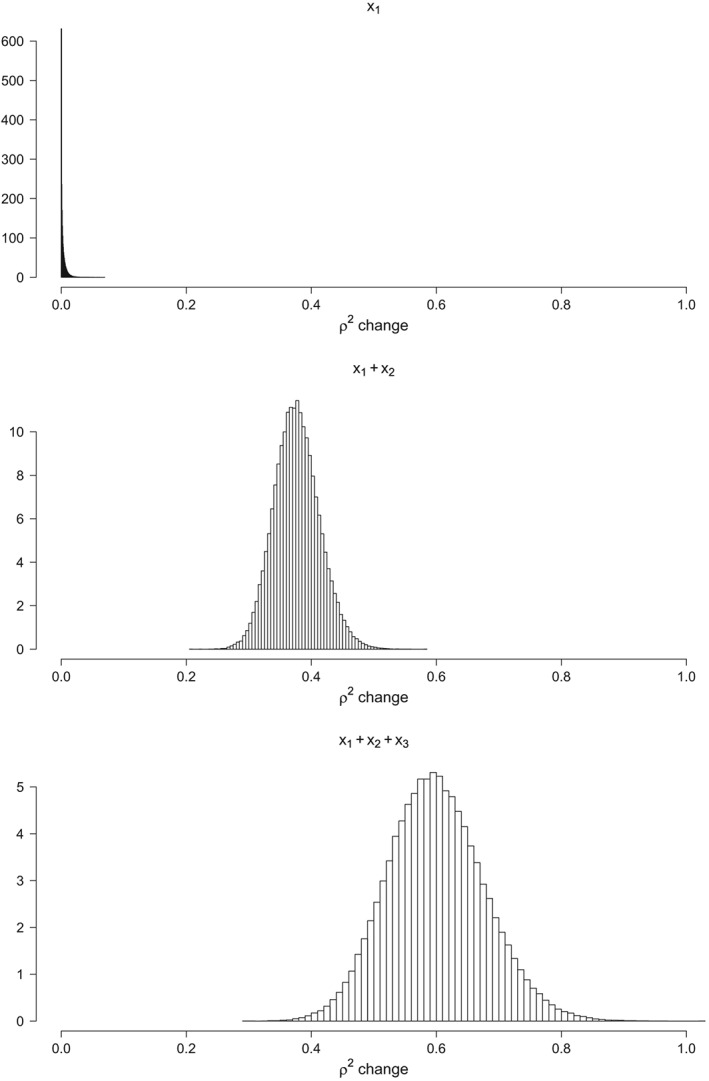
Histograms of 100,000 draws from the posterior distribution of ρ ^2^‐change, with the top panel showing the unique contribution of the first fixed factor (**x**_1_), the middle panel showing the joint contribution of both fixed factors (**x**_1_ and **x**_2_), and the bottom panel showing the joint contribution of all three factors (**x**_1_, **x**_2_, and x_3_), that is, $\rho_{y\;x}^{2}$

The unique contributions of each of the three factors are visualized in Figure 2 as a *ρ* ^2^‐change, using 100,000 draws from the posterior distribution of each of the semipartial correlations. That is, in Figure 2, we show the amount of variance that is uniquely explained by the first fixed factor in the top panel, by the first and second fixed factors in the middle panel, and, finally, by the contribution for all three factors combined in the bottom panel.

## 8 DISCUSSION

In this paper, we have shown that the theory on squared multiple correlation coefficients provides a useful framework for deriving ES measures for the ANOVA model, and we have analyzed several aspects that are commonly encountered in experimental designs. This allowed us to detail some expressions for the correlation coefficients and consequently clarify its underlying assumptions. As a result, we have shown that squared correlation measures encompass many of the existing ES measures that are now used for ANOVA, such as *ω* ^2^ and *η* ^2^, which are found to be special cases of the multiple correlation coefficient. Once the correlation expression is obtained, we note that it is easily evaluated using available software when the parameters of the design model are assumed fixed. In this manner, posterior interval estimates are simple to compute, which we have illustrated using two real‐data examples. Furthermore, we have shown in our examples how prior information can be included in the evaluation of the correlation coefficient and illustrated a Bayesian *ρ* ^2^‐change for the ANOVA model.

The analysis of the two‐way ANOVA design was complicated by the functional relation between the indicator variables for the main effects and the indicator variables encoding the interaction. We have solved this complication by combining two different approaches: one in which we only use the indicator variables encoding the interactions and one in which we only use the indicator variables that correspond to the main effects. In this manner, we were able to partial out the (unique) contribution of the main effects and the interaction in the particular case of uniform cell probabilities. Although not explicitly considered here, we believe that this approach will extend to situations involving more factors and interaction effects and to situations involving higher‐order interactions.

## ANALYTIC EXPRESSION FOR ${\tilde{\Delta }}^{T}{\tilde{\Gamma }}^{-1}\tilde{\Delta }$

Letting $\tilde{𝛑}={\left(\pi_{1},\;\text{…},\pi_{n-1}\right)}^{T}$ and $\tilde{D}=\text{diag}(\tilde{𝛑})$, we write the (*n* − 1) × (*n* − 1) matrix $\tilde{\Gamma }$ excluding the contribution of *x*_*n*_ as

$$\tilde{\Gamma }=\tilde{D}-\tilde{𝛑}{\tilde{𝛑}}^{T}.$$

To apply Equation (2), we require the inverse (Sherman & Morrison, 1950), that is,

$${\tilde{\Gamma }}^{-1}={\tilde{D}}^{-1}+\frac{{\tilde{D}}^{-1}\tilde{𝛑}{\tilde{𝛑}}^{T}{\tilde{D}}^{-1}}{1-{\tilde{𝛑}}^{T}{\tilde{D}}^{-1}\tilde{𝛑}}={\tilde{D}}^{-1}+\pi_{n}^{-1}11^{T}.$$

It is easy to verify that

$$\tilde{\Delta }=\tilde{D}b^{T},$$

where $\tilde{\Delta }=\text{Cov}(\tilde{x},\;y)$, $\tilde{x}={\left(x_{1},\;\text{…},\;x_{n-1}\right)}^{T}$, and $b={\left(b_{1},\;\text{…},\;b_{n}\right)}^{T}$ with $b_{i}=\beta_{i}-\sum_{i=1}^{n}\pi_{i}\beta_{i}=\beta_{i}-\beta$, such that

$$\begin{array}{ll} {\tilde{\Delta }}^{T}{\tilde{\Gamma }}^{-1}\tilde{\Delta } & =b\tilde{D}{\tilde{D}}^{-1}\tilde{D}b^{T}+\pi_{n}^{-1}b\tilde{D}11^{T}\tilde{D}b^{T}\\ & =b\tilde{D}b^{T}+\pi_{n}^{-1}b\tilde{𝛑}{\tilde{𝛑}}^{T}b^{T}\\ & =\overset{n-1}{\underset{i=1}{\sum }}\pi_{i}{\left(\beta_{i}-\beta \right)}^{2}+\pi_{n}^{-1}{\left(\overset{n-1}{\underset{i=1}{\sum }}\pi_{i}(\beta_{i}-\beta )\right)}^{2}\\ & =\overset{n-1}{\underset{i=1}{\sum }}\pi_{i}{\left(\beta_{i}-\beta \right)}^{2}+\pi_{n}^{-1}{\left(\overset{n-1}{\underset{i=1}{\sum }}\pi_{i}\beta_{i}-\overset{n-1}{\underset{i=1}{\sum }}\pi_{i}\beta \right)}^{2}\\ & =\overset{n-1}{\underset{i=1}{\sum }}\pi_{i}{\left(\beta_{i}-\beta \right)}^{2}+\pi_{n}^{-1}{\left(\beta -\pi_{n}\beta_{n}-(1-\pi_{n})\beta \right)}^{2}\\ & =\overset{n-1}{\underset{i=1}{\sum }}\pi_{i}{\left(\beta_{i}-\beta \right)}^{2}+\pi_{n}^{-1}{\left(\pi_{n}\beta -\pi_{n}\beta_{n}\right)}^{2}\\ & =\overset{n}{\underset{i=1}{\sum }}\pi_{i}{\left(\beta_{i}-\beta \right)}^{2}. \end{array}$$
